# Before or After: Evolving Neoadjuvant Approaches to Locally Advanced Non-Small Cell Lung Cancer

**DOI:** 10.3389/fonc.2018.00005

**Published:** 2018-01-23

**Authors:** Jennifer Lewis, Erin A. Gillaspie, Evan C. Osmundson, Leora Horn

**Affiliations:** ^1^Division of Hematology/Oncology, Department of Medicine, Vanderbilt University Medical Center, Nashville, TN, United States; ^2^Veterans Health Administration-Tennessee Valley Healthcare System, Geriatric Research Education Clinical Center, HSR&D Center, Nashville, TN, United States; ^3^Department of Thoracic Surgery, Vanderbilt University Medical Center, Nashville, TN, United States; ^4^Department of Radiation Oncology, Vanderbilt University Medical Center, Nashville, TN, United States

**Keywords:** neoadjuvant chemotherapy, induction chemotherapy, neoadjuvant chemoradiation, tri-modality, stage IIIA non-small cell lung cancer, mediastinal disease

## Abstract

The treatment of patients with stage IIIA (*N*2) non-small cell lung cancer (NSCLC) is one of the most challenging and controversial areas of thoracic oncology. This heterogeneous group is characterized by varying tumor size and location, the potential for involvement of surrounding structures, and ipsilateral mediastinal lymph node spread. Neoadjuvant chemotherapy, administered prior to definitive local therapy, has been found to improve survival in patients with stage IIIA (*N*2) NSCLC. Concurrent chemoradiation has also been evaluated in phase III studies in efforts to improve control of locoregional disease. In certain instances, a tri-modality approach involving concurrent chemoradiation followed by surgery, may offer patients the best chance for cure. In this article, we provide an overview of the trials evaluating neoadjuvant therapy in patients with stage IIIA (*N*2) NSCLC that have resulted in current practice strategies, and we highlight the areas of uncertainty in the management of this challenging disease. We also review the current ongoing research and future directions in the management of stage IIIA (*N*2) NSCLC.

## Introduction

An estimated 222,500 new cases of lung cancer are expected in the United States in 2017, of which approximately 80% will be non-small cell lung cancer (NSCLC) (1). Furthermore, approximately 15% of NSCLC cases will present with stage IIIA (*N*2) disease (2). This highly heterogeneous group is characterized by widely variable tumor sizes (sub-centimeter up to 7 cm), possible invasion of local structures, and microscopic or bulky ipsilateral mediastinal or subcarinal lymph node involvement. The degree of mediastinal lymph node involvement has significant prognostic implications. Patients with microscopic involvement have an estimated 5-year overall survival (OS) of 34%. However, OS falls to 11% when more than one lymph node station is involved with microscopic disease and 3–8% for patients with clinical lymph node involvement, seen radiographically, or including multiple stations (3).

Initial staging of lung cancer is performed *clinically* with radiographic studies evaluating the primary tumor size and the presence of enlarged lymph nodes. Such studies may not accurately reflect nodal status since microscopic disease would not be detectable by imaging studies alone. A pathologic evaluation of the mediastinum with EBUS or mediastinoscopy is paramount to accurate staging, allowing clinicians to determine the optimal strategy in care.

Due to the wide range of presentations within this group, defining the most effective treatment approach has been historically challenging. In the 1970s, the overall cure rate for lung cancer was estimated to be 25% following resection (4). Surgeons at the time noted long-term survivals and an increase in the 5-year survival of up to 30–40% with surgery in a subset of patients with stage IIIA NSCLC with peripheral tumors and microscopic *N*2 disease (5–7). Unfortunately, outcomes were complicated by high rates of locoregional failure and distant recurrence following resection, which led investigators to consider a neoadjuvant treatment strategy for this group of NSCLC patients.

Neoadjuvant, or induction, therapy is defined as therapy administered prior to definitive local treatment (8). Neoadjuvant chemotherapy in patients with early and advanced stage NSCLC was first used in the 1950s, when investigators employed mitomycin C and chromomycin A3 in a “long-term intermittent schedule,” in which the first cycle was started prior to surgery (4). A dose of mitomycin C was even “infused directly into the pulmonary vein draining the tumor” during surgery, with the remainder of the cycle delivered post-operatively. Patients then continued on 4-week cycles for up to 3 years after surgery (4). Today, the neoadjuvant therapeutic strategy remains one of the most hotly debated topics among thoracic oncology specialists. The theoretical benefit of neoadjuvant therapy includes earlier treatment of micrometastatic disease, reduction in tumor burden, evaluation of tumor sensitivity *in vivo*, prevention of tumor seeding at the time of surgery, and possible improved compliance with therapy (9). In this article, we will review the literature on the use of neoadjuvant systemic therapy for stage IIIA (*N*2) NSCLC, focusing on phase III trials as well as areas of further research.

## Neoadjuvant Chemotherapy

The benefit of neoadjuvant chemotherapy was first suggested by multiple single-arm, and later randomized, phase II studies. These trials demonstrated 3-year survival rates as high as 34% and a median survival of up to 23 months in patients with stage IIIA (*N*2) NSCLC treated with neoadjuvant chemotherapy followed by resection, Table 1 (10–27). As a result, several randomized-controlled trials were designed to evaluate these findings.

**Table 1:** Neoadjuvant chemotherapy phase II trials.

Reference	Chemotherapy	*N*	*N*2	ORR (% total)	Surgery (% total)	R0 (% surgery)	PCR (% total)	Survival
Martini et al. (10)	MVP	41	41	73	68	75	20	3 years 34%, 3 years 54% (R0),
Vokes et al. (11)	EVP	27	NR	48	15	NR	0	Median 8 months
Pujol et al. (12)	EPI	33	31	70	61	90	15	18 months 30%, median 10 months
Burkes et al. (13)	MVP	39	39	64	56	82	8	3 years 26%, median 19 months
Martini et al. (14)	MVP	136	136	77	84	78	14	3 years 28%, median 19 months; 3 years 41% (R0)
Darwish et al. (15)	PE	46	46	80	72	85	9	2 years 53%, median 25 months
Sugarbaker et al. (16)	VP	74	74	NR	85	37	0	3 years 23%, 3 years 46% (R0)
Elias et al. (17)	P, 5-FU	34	34	65	82	75	18	Median 18 months
van Zandwijk et al.^a^ (18)	GP	47	47	70	NS	NS	NS	Median 19 months
Betticher et al. (19)	DP	90	90	66	83	48	16	EFS 15 months, median 33 months
O’Brien et al.^a^ (20)	CT	52	52	64	NS	NS	NS	1 year 68%, median 21 months
De Marinis et al. (21)	GTP	49	49	74	59	93	16	1 year 85%, median 23 months
Cappuzzo et al. (22)	GP	129	88	62	31	95	2	1 year 74%, median 19 months
Burkes et al. (23)	MVP or VP	65	65	68	72	75	5	1 year 66%, median 19 months; 5 years 29%
Biesma et al.^a^ (24)	DP	46	46	39	NS	NS	NS	1 year 65%, median 16 months
Garrido et al. (25)	GDP	136	69	53	66	69	6	3 years 37%, median 16 months; 5 years 41% (R0)
Chaft et al. (26)	Bev, DP + Bev	50	NR	40	88	82	NR	3 years 64%
Ou et al. (27)	C, Pem, Bev	42	36	42	74	71	NR	1 year 56%, median EFS 15.4 months

*Bev, bevacizumab; C, carboplatin; D, docetaxel; EFS, event-free survival; E, etoposide; 5-FU, 5-fluorouracil; G, gemcitabine; I, ifosfamide; M, mitomycin-C; NR, not reported; NS, not significant; ORR, overall response rate P, cisplatin; V, PCR, pathologic complete response; Pem, pemetrexed; T, paclitaxel; vinblastine/vindesine*.

*^a^Patients in this trial were randomized to surgery or radiation after induction chemotherapy as part of the EORTC 08941*.

The first published randomized-controlled trial was a small, single-institution phase III trial performed at the National Cancer Institute. Patients with stage IIIA NSCLC, biopsy-proven *N*2 disease, were randomized to surgery alone followed by radiation or two cycles of neoadjuvant chemotherapy with cisplatin and etoposide followed by surgery (28). Patients in the chemotherapy arm with a response, defined radiographically by CT, at the time of surgery underwent an additional four cycles of cisplatin and etoposide post-operatively. Patients without a response to neoadjuvant chemotherapy received post-operative radiation to a total dose of 54–60 Gy. Twenty-seven patients were randomized over 4 years, and the median follow-up period for the chemotherapy arm was 29.9 and 34.9 months for the surgery alone arm. The overall response rate to chemotherapy was 62% (8 of 13 patients). There was no significant difference between the rates of R0 resections (85% in each arm) or types of surgical procedures used (pneumonectomy, lobectomy, etc.) in each arm of the study. There were also no post-operative deaths, which was likely attributable to selection bias and the center at which this trial was conducted. There was a trend toward improved survival in patients treated with neoadjuvant chemotherapy, 28.7 versus 15.6 months. In addition, the disease-free interval was longer in the chemotherapy arm (12.7 versus 5.8 months), and the recurrence rate was also lower in the chemotherapy arm (92 versus 73%) (28). This trial provided preliminary data, but was not conclusive due to its slow rate of accrual and small sample size, an inherent limitation of single-institution trials.

In a second phase III trial, investigators compared neoadjuvant chemotherapy with mitomycin, ifosfamide, and cisplatin every 21 days for three cycles followed by surgery versus surgery alone in 60 patients with stage IIIA NSCLC (29). The trial was terminated early when an interim analysis at 24 months found that neoadjuvant chemotherapy was associated with a significant improvement in median survival, 26 months versus 8 months. Although this was a dramatic improvement, critics point out the higher percentage of patients with tumors harboring KRAS mutations (42 versus 15%), a negative prognostic factor, in the control arm that may have biased the study (8).

A few months later, the results of a similar randomized-controlled trial involving 60 patients with stage IIIA NSCLC were published. This trial compared neoadjuvant chemotherapy with cyclophosphamide, etoposide, and cisplatin for three cycles followed by surgery and an additional three cycles of chemotherapy post-operatively in responders versus surgery alone. Similarly, this study was terminated early when an interim analysis demonstrated that 35% of patients had a radiographic response to neoadjuvant chemotherapy. Moreover, there was an even greater difference in survival with a median survival of 64 months in the chemotherapy arm compared to 11 months in the surgery only arm. The estimated 2- and 3-year survivals were 60 and 56%, respectively, for the patients who received chemotherapy compared to 25 and 15% for those who received surgery alone (30).

A larger randomized phase III trial conducted by the French Thoracic Cooperative Group also evaluated the role of neoadjuvant chemotherapy in patients with NSCLC. This trial randomized 355 patients with stage IB–IIIA to surgery alone or two cycles of induction chemotherapy with mitomycin, ifosfamide, and cisplatin followed by surgery and then two additional cycles of chemotherapy. Patients who were found to have pT3 or pN2 disease received post-operative radiation to a total dose of 60 Gy. Median survival was 37 months in the chemotherapy plus surgery arm compared to 26 months in the surgery alone arm. The 3-year survival (52 versus 41%) also favored the bi-modality treatment approach of neoadjuvant chemotherapy followed by surgery (31).

These four randomized-controlled trials demonstrated a substantial survival advantage, supporting the use of neoadjuvant chemotherapy for patients with stage IIIA NSCLC undergoing surgical resection. Since the publication of these studies, additional trials have been performed. A meta-analysis of 12 randomized studies from 1995 to 2005, some of which used more modern chemotherapy regimens, also suggests a survival advantage for neoadjuvant chemotherapy in NSCLC. However, many of these trials were small and included patients with early stage disease as well as stage IIIA (*N*2) (32). A logical next question would be, what is the best neoadjuvant chemotherapy regimen to use?

## Neoadjuvant Chemotherapy Choice

Many phase II trials have assessed the efficacy of various chemotherapy combinations. Platinum-based neoadjuvant regimens have consistently demonstrated the highest overall response rates ranging from 50 to 70% depending on the combination, Table 1 (10–27). Phase II studies have also demonstrated increased rates of resection with the use of neoadjuvant platinum-based regimens, albeit with the usual caveats of institutional bias and surgical capabilities at the sites where the trials were conducted (7). Neoadjuvant cisplatin and etoposide with or without radiation is perhaps the most studied regimen as we discuss in the trials below (15, 33–38). However, randomized phase II and phase III studies have also evaluated more modern agents, including cisplatin–vinorelbine, cisplatin–gemcitabine, cisplatin–docetaxel, and cisplatin–pemetrexed (18, 24, 39–41).

In general, carboplatin and paclitaxel is an attractive chemotherapy combination for the treatment of NSCLC due to its more favorable toxicity profile. This regimen has been evaluated in the neoadjuvant setting for NSCLC in several clinical trials, including a phase II trial conducted by the European Organization for Research and Treatment of Cancer (EORTC) in patients with stage IIIA (N2) disease. This trial demonstrated a response rate of 64%, median survival of 20.5 months, and an estimated 1-year survival of 68.5% in patients treated with carboplatin and paclitaxel (20). Randomized phase III trials have also employed this regimen, such as the S9900 trial that randomized patients with stage I–IIIA NSCLC to three cycles of neoadjuvant chemotherapy or to surgery alone. Of note, this trial excluded patients with single-station *N*2 disease. The overall response rate to neoadjuvant chemotherapy was 41% and the OS improved from 46 months in the control arm to 74 months in the neoadjuvant chemotherapy arm (42). A large, randomized European phase III trial (NATCH) demonstrated a 53.3% overall response rate for neoadjuvant carboplatin and paclitaxel in patients with early stage NSCLC. Lastly, other chemotherapy combinations utilizing carboplatin have been studied. A small phase II trial found that carboplatin, pemetrexed, and bevacizumab are safe in the neoadjuvant setting with a response rate of 45% and median survival of 37 months (26).

The major advantage of using a neoadjuvant platinum-based chemotherapy is the 12% relative survival benefit or 5% OS improvement in 5 years (41). Another benefit of neoadjuvant chemotherapy is improved compliance. One randomized phase III trial that illustrates this well is the NATCH trial, which compared adjuvant versus neoadjuvant chemotherapy in patients with early stage NSCLC. In this trial, 97% of patients started neoadjuvant chemotherapy and 90% of patients completed neoadjuvant chemotherapy while only 62% started adjuvant chemotherapy and 61% completed adjuvant chemotherapy (43). Perhaps most important, neoadjuvant chemotherapy is not associated with increased post-operative complications or death (44–46).

## Neoadjuvant Chemotherapy versus Neoadjuvant Chemoradiation

Efforts to improve locoregional control, pathologic response, and resectability led investigators to ask the question of whether the addition of radiation would increase survival compared to induction chemotherapy alone. Several phase II trials, summarized in Table 2 (35–38, 47–55), were designed to evaluate the role of neoadjuvant concurrent chemoradiation. Interestingly, five of these studies demonstrated complete pathologic responses of 15–26% and resection rates as high as 80–90%. The results of these studies prompted the development of several randomized phase III trials.

**Table 2:** Neoadjuvant concurrent chemoradiation phase II trials.

Reference	Chemoradiation	*N*	*N*2	ORR (% total)	Surgery (% total)	R0 (% surgery)	PCR (% total)	Survival
Taylor et al. (47)	P, 5-FU + 40 Gy	64	50	58	61	NR	14	1 year 61%, median 16 months
Pincus et al. (48)	PE, 5-FU + 40 Gy	31	NR	74	39	100	19	2 years 33%, median 15 months
Faber et al. (49)	P, 5-FU or PE, 5-FU + 40 Gy	85	62	NR	71	NR	20	3 years 40%, median 37 months
Recine et al. (50)	PE, 5-FU + 40 Gy	64	NR	84	36	100	14	3 years 30%, median 13 months; 3 years 69% (resection)
Strauss et al. (51)	VP, 5-FU + 30 Gy	41	33	51	61	96	10	1 year 58% median 16 months
Palazzi et al. (35)	PE + 40 Gy	43	21	70	30	92	7	1 year 58%, 2.5 years 21%
Weiden et al. (52)	P, 5-FU + 30 Gy	85	68	56	52	66	9	Median 13 months
Albain et al. (37)	PE + 45 Gy	126	75	59	71	98	15	2 years 37% (*N*2); median 13 months (*N*2)
Favaretto et al. (36)	PE + 51.2 Gy	39	NR	64	51	NR	8	3 years 18%, median 16 months
Choi et al. (53)	VP, 5-FU + 42 Gy	42	42	74	93	87	10	Median 25 months, 5 years 37%
Eberhardt et al. (38)	PE + 45 Gy	94	56	64	66	81	26	Median 20 months (IIIA); 4 years 31% (IIIA) 46% (R0)
Thomas et al. (54)	ICE + 45 Gy	54	25	69	74	85	13	2 years 40%, median 20 months
D’Angelillo et al. (55)	GP + 50.4 Gy	50	29	80	82	88	26	3 years 40%, median 22 months

*C, carboplatin; E, etoposide; 5-FU, 5-fluorouracil; G, gemcitabine; I, ifosfamide; NR, not reported; ORR, overall response rate; P, cisplatin; PCR, pathologic complete response; V, vinblastine/vindesine*.

The German Lung Cancer Cooperative Group (GLCCG) was a multi-institutional, randomized phase III trial that enrolled 558 patients with stage IIIA/IIIB NSCLC. The investigators included marginally resectable stage IIIA/IIIB patients, postulating that neoadjuvant treatment could downstage these tumors, rendering them resectable. Patients were randomized to receive either neoadjuvant chemotherapy with cisplatin and etoposide followed by concurrent chemoradiation and resection or neoadjuvant chemotherapy followed by resection and post-operative radiation (33). Neoadjuvant radiation was administered in a hyperfractionated schedule, given twice daily (1.5 Gy per fraction), to a total dose of 45 Gy with weekly carboplatin and vindesine. Notably, all patients in the control arm also received post-operative radiation independent of margin status. A total dose of 54 Gy (1.8 Gy per fraction) was administered in the setting of negative margins (R0 resections) and up to 68.4 Gy if margins were positive or if tumors were deemed unresectable. Responses to neoadjuvant treatment were assessed using CT of the chest, abdomen, and brain. Only patients without disease progression proceeded to concurrent radiation and resection. The primary end point was progression-free survival (PFS) and the secondary endpoints included OS and the proportion of patients undergoing surgery. Of the patients who underwent surgery, the proportions of patients with negative resection margins, complete resections, histopathologic response (tumor regression of >90%), and mediastinal down-staging were evaluated as secondary endpoints. Fifty-four percent of patients in the concurrent chemoradiation arm and 59% of patients in the induction chemotherapy arm underwent resection with 37 and 32% undergoing complete resections in the concurrent chemoradiation arm and induction chemotherapy arm, respectively. There was a significant difference in mediastinal down-staging (*N*2–3 to *N*0–1) (*p* = 0.02) and histopathologic response (*p* = <0.0001), favoring the chemoradiation arm (33). There was no difference in PFS or OS between the two groups even when evaluating only those patients who had a resection. However, compared to patients who underwent an incomplete resection, patients who had a complete resection had longer median, 1-, 3-, and 5-year PFS (median 28.7 versus 21.1 months) and longer OS (median 50.6 versus 20.4 months). More patients who underwent a complete resection were found to have mediastinal down-staging, and this was the only independent predictor of improved PFS and OS on multivariate analysis [HR 2.11 (1.23–3.62), *p* = 0.007] (33). Finally, 35% of patients in both arms of the study underwent a pneumonectomy, and treatment-related mortality was higher among patients who received neoadjuvant chemoradiation (14%) compared to induction chemotherapy (6%) (33).

Since publication of the GLCCG trial, several retrospective and other studies demonstrate a lack of improved survival with the addition of neoadjuvant radiation (56–58). A systematic review and meta-analysis of pooled data from 156 patients with stage IIIA NSCLC from seven randomized and retrospective studies comparing neoadjuvant chemotherapy to neoadjuvant concurrent chemoradiation found no survival benefit with the addition of radiation (HR 0.93, 95% CI: 0.54–1.62, *p* = 0.81) (56). Also, a large, retrospective study of 1076 patients with stage IIIA NSCLC, most with *N*2 disease (*N* = 903), from the National Cancer Database found no difference in survival for patients treated with neoadjuvant chemotherapy compared to neoadjuvant concurrent chemoradiation. However, patients treated with concurrent chemoradiation were found to have decreased residual nodal disease (57). It is important to keep in mind that the studies used in these pooled analyses employed a heterogeneous mix of treatment regimens, some with outdated radiation techniques.

One of the most intriguing findings of the GLCCG trial was the increased mediastinal down-staging and histopathologic tumor regression on final pathology in patients who underwent a complete resection after treatment with neoadjuvant chemoradiation compared to chemotherapy. Although the GLCCG did not demonstrate an improvement in OS, a small retrospective analysis of 92 patients with stage IIIA (*N*2) NSCLC found a trend toward improved survival in patients with mediastinal down-staging and had complete resections after neoadjuvant chemotherapy. There was also a 5-year survival benefit for single-station *N*2 compared to multi-station disease discovered at the time of initial mediastinoscopy (37 versus 7% 5-year survival, *p* = < 0.005) (59). Other retrospective studies show improved outcomes in patients with increased nodal clearance after neoadjuvant chemoradiation. One study found increased disease-free survival in patients with mediastinal complete pathologic response after neoadjuvant chemoradiation (58). Another study even found an increase in 5-year survival as high as 47% in patients with a partial or complete nodal pathologic response to chemoradiation (58, 60). Thus, there is evidence that mediastinal down-staging is associated with improved outcomes. In fact, complete nodal pathologic response is widely considered as a surrogate for a favorable prognosis (34, 60).

There is also indirect evidence that neoadjuvant chemoradiation may provide improved outcomes compared to induction chemotherapy followed by resection. The phase III EORTC 08941 (discussed in the Section “Neoadjuvant Tri-modality Therapy”) compared induction chemotherapy followed by surgery versus sequential chemotherapy and radiation. Patients who received radiation had a similar OS and PFS with lower morbidity and mortality (2). In unresectable stage III NSCLC, concurrent chemoradiation has been found to be superior to sequential chemotherapy and radiation (61) (discussed in the Section “Sequential versus Concurrent Chemotherapy and Radiation”). Taken together, this suggests that neoadjuvant concurrent chemoradiation may be superior over induction chemotherapy alone followed by surgery. However, we emphasize this comparison has not been adequately addressed in a large phase III trial to date.

The results from the GLCCG trial highlight several key challenges when designing trials for patients with stage IIIA (*N*2) NSCLC. One is the definition of resectability—over 60% of patients had stage IIIB NSCLC (T4*N*2 or *N*3 disease) that is generally considered unresectable. Restaging after neoadjuvant treatment can vary between institutions, and details regarding restaging in the GLCCG are unclear. Reassessment of the mediastinum prior to surgery does not appear to have been performed aside from chest CT. This may have accounted for the higher than expected rate of incomplete resections at the time of surgery. Another challenge in designing trials for patients with stage IIIA (*N*2) NSCLC is the coordination between treatment modalities. Among patients in the GLCCG interventional arm treated with concurrent chemoradiation arm, only 54% ultimately went on to surgery (37% with R0 resection) and the remaining received an additional 24 Gy that was resumed after a 4- to 6-week break for response assessment. It is well documented that prolonged radiotherapy treatment breaks lead to worse outcomes (62, 63). In addition, trials evaluating treatment strategies for patients with stage IIIA (*N*2) NSCLC have been hampered by slow accrual rates, impeding the trials’ ability to stay relevant. For example, the radiation techniques used in the GLCCG became outdated over the course of the trial. Neither the hyperfractionated radiation schedule nor the target volumes employed are standard of care at this time (64). Furthermore, all patients in the control neoadjuvant chemotherapy arm received post-operative radiation regardless of pathological nodal involvement, margin status, or extent of resection, a practice that is controversial (65, 66). Finally, many trials evaluating treatment for patients with stage IIIA (*N*2) NSCLC have also been complicated by high pneumonectomy rates, which are associated with worse outcomes (33, 34).

## Neoadjuvant Concurrent Chemotherapy Choice

What is the best neoadjuvant chemotherapy regimen to use when treating patients concurrently with radiation? A platinum-based doublet is the recommended regimen. Several combinations with radiation have been evaluated in phase II neoadjuvant trials. As seen in Table 2 (35–38, 47–55), older cisplatin-containing regimens with 5-fluorouracil, cyclophosphamide, doxorubicin, and vinblastine demonstrate response rates ranging from 50 to 70% and 15–20 month median survival times. Cisplatin and etoposide with radiation has been used extensively in phase II and phase III trials and demonstrates an overall response rate of 60–70% (28, 33–38). This regimen has been preferred among investigators for its manageable outpatient administration and ability to administer an upfront therapeutic-dose concurrently with radiation. Although not evaluated in the neoadjuvant setting, many oncologists use more modern regimens, such as carboplatin/paclitaxel, cisplatin, or carboplatin/pemetrexed, concurrently with radiation. These regimens have been found to have good efficacy and tolerability in the treatment of unresectable NSCLC (67–69). Cisplatin/gemcitabine is a modern, efficacious regimen that has been evaluated in the neoadjuvant setting (55). However, this regimen is highly toxic when combined with radiation and is not commonly used.

Trials evaluating neoadjuvant concurrent chemoradiation with immunotherapy are currently ongoing (nivolumab, pembrolizumab) (70). Because pneumonitis is a known potential side-effect of both immunotherapy and radiotherapy, chemotherapy regimens associated with lower rates of pneumonitis may ultimately become more favorable in this setting. The PROCLAIM trial (69) and a well-conducted Chinese trial (71) that was stratified by factors known to be associated with radiation pneumonitis [percentage of lung volume that receives 20 Gy or more (V20), diffusion capacity of carbon monoxide (DLCO), and gross tumor volume] have shown lower rates of pneumonitis for cisplatin/etoposide versus cisplatin/pemetrexed or carboplatin/paclitaxel. In the PROCLAIM trial, pneumonitis of any grade was significantly higher in the patients treated with cisplatin/pemetrexed versus cisplatin/etoposide (17 versus 10.7%) although there was no difference in Grade 3–4 pneumonitis between treatment arms (69). In the Chinese trial, grade 2 or greater radiation pneumonitis was more frequent in the carboplatin/paclitaxel arm (33.3%) versus cisplatin/etoposide arm (18.9%), although there were no significant differences in rates of grade 3 or greater pneumonitis between arms (71). Nonetheless, chemotherapy regimens that are less likely to be associated with pneumonitis may become more important in the future if immunotherapy is also incorporated into concurrent chemoradiation treatment plans.

The National Comprehensive Cancer Network issued a provider survey to its members in 2010 and found that approximately 50% of providers use neoadjuvant chemotherapy while the other 50% use neoadjuvant concurrent chemoradiation therapy more often (64). The authors of this paper prefer cisplatin and etoposide with radiation if the patient has a good performance status and no significant comorbidities given its extensive use in clinical trials and the data supporting this regimen.

## Sequential versus Concurrent Chemotherapy and Radiation

Sequential chemotherapy and radiation has been studied extensively in patients with stage III NSCLC. A meta-analysis of seven phase III trials comparing concurrent with sequential chemotherapy and radiation in patients with stage III NSCLC included 1,205 patients, 61% with stage IIIB and 37% with stage IIIA. Median follow-up was 6 years (61). In this pooled analysis, a significant OS benefit (HR 0.84; 95% CI: 0.74, 0.95; *p* = 0.004) was found for patients who were treated with concurrent chemoradiation compared to sequential therapy. The absolute benefit was found to be 5.7% at 3 years and 4.5% at 5 years. There was also a trend toward an improved PFS with a hazard ratio of 0.90 (95% CI: 0.79, 1.01; *p* = 0.07) and a decrease in locoregional progression in the concurrent chemoradiation group (61). There were more toxicities associated with concurrent chemoradiation, particularly esophagitis (61). Nonetheless, sequential chemotherapy and radiation is not routinely considered standard of care for stage IIIA NSCLC, including patients with *N*2 involvement. However, in patients with poorer performance status, who would not tolerate concurrent chemoradiation, sequential therapy is a potential treatment option.

## Neoadjuvant Tri-Modality Therapy

Two multicenter randomized-controlled trials assessed whether resection was necessary following neoadjuvant chemotherapy with or without radiation in patients with stage IIIA NSCLC. EORTC 8941 was a phase III European study that assessed whether surgery is superior to radiation following a radiologic response to induction chemotherapy with a platinum-based regimen in patients with unresectable stage IIIA (N2) disease (2). OS was the primary endpoint. Secondary endpoints included PFS and safety. This trial randomized 332 patients over 10 years. The overall response rate to induction chemotherapy was 61% (2). Compliance among the patients assigned to receive radiation was poor at 55%, with major protocol deviations that included, but were not limited to, inconsistent total radiation dose and/or fractionation schemes, and timing of radiotherapy administration (72). Of the patients who were assigned to undergo surgery, 92% underwent a procedure, but only 50% of patients had a complete resection and 47% of these required a pneumonectomy. PFS and OS were similar in both groups; median PFS was 9 months in the surgery arm versus 11.3 months in the radiation arm and median OS was 16.4 months in the surgery arm versus 17.5 months in the radiation arm. Of the patients who underwent resection, those who had a lobectomy, complete resection, and pathological clearance of the mediastinal lymph nodes did better than those who underwent a pneumonectomy, an incomplete resection, or did not have pathologically clearance in mediastinal lymph nodes. The mortality rate associated with pneumonectomy was 7% compared to 4% overall surgical mortality (2). The strengths of this trial include the large, multicenter population and the requirement for pathological confirmation of *N*2 disease. However, changes in the staging system (PET scan and brain imaging were not performed) may have allowed the inclusion of patients with more advanced disease (64). In addition, the outcome of all patients initially enrolled in the trial is not well described, as only the outcomes of patients who were randomly assigned to therapy are reported. Finally, outdated radiotherapy techniques, slow accrual rate, poor compliance, inconsistency of specialized, thoracic surgeons, and the use of sequential chemotherapy and radiation as definitive therapy in the control arm are potential limitations of this trial (64).

The North American Intergroup 0139 (INT 0139) trial similarly sought to evaluate the potential role of surgery as part of a tri-modality treatment strategy for patients with stage IIIA (*N*2) NSCLC (34). This trial was performed in North America and randomized patients with resectable stage IIIA and pathologically confirmed *N*2 disease to receive either concurrent chemotherapy with cisplatin and etoposide and radiation to 45 Gy followed by resection or concurrent chemotherapy with cisplatin and etoposide and radiation to 45 Gy followed by definitive radiation to 61 Gy, administered in an uninterrupted schedule (29). If patients randomized to the surgery arm did not progress radiographically by CT after completing neoadjuvant chemoradiation, they proceeded to resection. Both groups received two cycles of post-operative chemotherapy with cisplatin and etoposide either after surgery or with completion of definitive radiation. The primary end point was OS, and secondary endpoints were PFS, toxicity, and patterns of failure. The two study arms were well balanced and enrolled 429 patients over 7 years. The majority of patients had biopsy-proven single-station *N*2 lymph node involvement. Of the patients found to be eligible for surgery, 81% underwent thoracotomy, 71% had complete resections, and 55% completed consolidation chemotherapy. Of the patients randomized to definitive radiation, 92% continued radiation without a break in treatment. The median follow-up was 22.5 months. OS did not differ between the two treatment arms, although there was a late trend toward improved OS in the tri-modality treatment arm as well as an increased PFS in the patients treated with tri-modality compared to those who received definitive radiation (12.8 versus 10.5 months). The greatest benefit was seen in patients having a pathologic response (*N*0) at the time of surgery with a median survival of 34.4 months. Fifty-four patients underwent pneumonectomy with a concerning high mortality rate of 26%. The most common grade 3 or 4 toxicity was neutropenia in both treatment groups. Grade 3 or 4 esophagitis and pneumonitis were more common toxicities in patients who underwent definitive radiation compared to surgical resection (34). The investigators performed an unplanned, exploratory, matched subset analysis that suggested tri-modality therapy could benefit patients if a lobectomy and complete resection are possible. In this population, the median survival time was 33.6 months in the tri-modality group compared with 21.7 months in matched patients treated with definitive concurrent chemoradiation (*p* = 0.002). It remains unclear whether this differs significantly from the 28.7-month median survival of patients with stage III NSCLC randomized to the control arm of the recently published RTOG 0617 trial who were treated with concurrent chemoradiation alone (60 Gy), especially given that 34% had more advanced, stage IIIB disease (73). Although positron emission tomography (PET), which can assist in the detection of regional and distant disease, was not used in the INT 0139 trial, the patient population was otherwise rigorously staged with mediastinoscopy and surgical nodal sampling. Treatment adherence was excellent in both arms with high compliance and resection rates compared to other phase III trials.

Criticisms of INT0139 include the incomplete accrual rate, an underpowered subset analysis suggesting a tri-modality therapy advantage, and a very high mortality rate among patients who underwent pneumonectomy (64). It is also important to point out that the radiation dose administered concurrently with chemotherapy in the surgical arm is considered sub-therapeutic (45 Gy) in the definitive setting, and nearly one in five patients enrolled on this arm did not ultimately undergo thoracotomy. This study, such as the GLCCG trial, highlights the challenges with committing to tri-modality therapy without risking sub-therapeutic treatment or prolonged, detrimental breaks in treatment.

More recently, the results of RTOG 02-29, a phase II trial evaluating therapeutic-dose neoadjuvant chemoradiation in stage III (*N*2 or *N*3, supraclavicular disease excluded) show both the safety and feasibility of delivering neoadjuvant radiotherapy regimens to a dose of 61.2 Gy. In addition to high rates of mediastinal nodal clearance (63%), this regimen eliminates the potential for delivery of sub-therapeutic radiotherapy and/or radiotherapy treatment breaks in patients who do not ultimately undergo complete resection (74).

Despite its limitations, INT0139 represents the strongest evidence to date for the use of tri-modality treatment. Its subset analysis suggests that patients with potentially resectable disease using a lobectomy may benefit from neoadjuvant tri-modality therapy with concurrent chemoradiation followed by surgery. In this setting, two local therapies (radiation and surgery) may downstage microscopic nodal disease prior to resection, leading to improved outcomes. Therefore, early evaluation by a thoracic surgeon is important in order to identify patients with locally advanced NSCLC who may benefit from tri-modality treatment.

## Timing of Surgery

A practical concern with a neoadjuvant treatment strategy is the potential for delay of definitive local therapy, which has been associated with worse survival (75). For this reason, it is crucial that patients are evaluated by a thoracic surgeon early, not only to determine resectability but to also allow for timely planning of restaging and resection, ideally within 6 weeks from completion of neoadjuvant therapy. A large, retrospective study of 1,623 patients in the National Cancer Database with stage IIIA NSCLC who were treated with neoadjuvant concurrent chemoradiation from 2004 to 2012 found a statistically significant decline in survival when surgical resection occurred greater than 6 weeks from the completion of neoadjuvant therapy (76). Examining the data more closely, the investigators compared survival times for patients at 0–3, 3–6, 6–9, and 9–12 weeks after completion of neoadjuvant therapy. Although it was *not* statistically significant, there was a trend toward reduced survival when extending surgery to 3–6 weeks compared to 0–3 weeks (45.2 versus 60.7 months, respectively, *p* = 0.107 in multivariate analysis). The survival difference between 0–3 and 6–9 weeks *was* statistically significant (*p* = 0.043 in multivariate analysis). Comparing the survival difference for 3–6 weeks and 6–9 weeks, this was a very small difference (45.2 versus 44.1 months); in fact, the Kaplan–Meier curves for weeks 3–6 and 6–9 touched in some areas (76). Therefore, it may be optimal to plan surgery even earlier than six weeks and closer to 3–4 weeks post-neoadjuvant therapy, assuming patients have recovered from their local therapy.

## Future Directions

Cytotoxic chemotherapy may not be the only systemic therapy used in future neoadjuvant regimens for stage IIIA NSCLC. Considerable attention has focused on targeted therapy and immune checkpoint inhibitors as potential neoadjuvant therapies for locally advanced NSCLC. A small phase II trial compared erlotinib to carboplatin–gemcitabine in 24 patients with resectable, EGFR mutant, stage IIIA (*N*2) NSCLC and found an overall response of 38% for erlotinib compared to 25% for chemotherapy, although there was no survival benefit for erlotinib (77). In another small, single-arm phase II study, investigators administered erlotinib for 3 weeks prior to surgery in 60 patients with early stage NSCLC. Of note, EGFR testing was obtained on surgical specimens (78). A subset of 15 Asian, female never smokers with non-squamous histology, were analyzed separately as a cohort more likely to harbor an EGFR mutation. The response rate was low overall (5% by RECIST on CT, 27% metabolic response by PET), and 12% of the total population was found to have an EGFR mutation. The response rate increased to 34% in the Asian female subset, 17% of which were found to be EGFR mutated. Furthermore, 23% of patients who underwent resection had more than 50% necrosis at the time of pathology review. Toxicities were tolerable and included rash and diarrhea, which are typical of EGFR tyrosine-kinase inhibitors (78). Additional studies using chemotherapy and/or targeted therapy in the neoadjuvant setting have closed due to poor accrual (79).

As in many areas in oncology today, there are multiple ongoing trials evaluating neoadjuvant immunotherapy, including atezolizumab, pembrolizumab, nivolumab with or without ipilimumab, durvalumab as well as combination checkpoint inhibitors and chemotherapy (79). We will have to await the maturation of these and future clinical trials before determining the role of neoadjuvant targeted therapy and immunotherapy.

Aside from novel therapeutics, other strategies to improve outcomes in patients with stage IIIA (*N*2) are being studied, including the role of PET scan in assessment of response. Fluorine-18 fluorodeoxyglucose (FDG)-PET/CT is a standard of care for the staging of patients initially diagnosed with NSCLC (80). A meta-analysis has shown that increased standardized uptake value (SUV) of the primary tumor is a poor prognostic factor in NSCLC (81). A retrospective review has already shown that patients with stage II NSCLC treated with neoadjuvant chemotherapy who have a greater than 50% reduction in SUV on PET scan demonstrate a trend toward improved survival compared to patients with less than a 50% reduction in SUV (82). A prospective study (*N* = 79), including 25 patients with stage IIIA (*N*2) NSCLC demonstrated that FDG uptake in the mediastinal lymph nodes after three cycles of neoadjuvant chemotherapy was associated with a twofold higher risk of mortality whereas repeat CT was not a predictor of survival (83). In fact, a 35% decrease of FDG uptake after one cycle of neoadjuvant chemotherapy discriminated responders from non-responders (83). Taking these findings further, a recent phase II study assessed the timing of treatment switch to optimize response rates (84). In this phase II study, 40 patients with resectable stage IB to IIIA NSCLC received neoadjuvant chemotherapy with a platinum-based doublet (carboplatin or cisplatin plus gemcitabine or pemetrexed). A PET scan was performed after two cycles. If the SUV decreased by at least 35%, patients continued on the initial chemotherapy regimen, but if the SUV did not decrease by at least 35%, the patients were switched to a different chemotherapy regimen (docetaxel–vinorelbine). Sixty-seven percent of patients who were switched to a different regimen had a metabolic response on subsequent PET scan (84). These studies are small, and PET scan responses as part of the strategy in the neoadjuvant treatment for stage IIIA (*N*2) NSCLC have not been tested in randomized phase III trials. However, we may consider metabolic responses by PET scan after neoadjuvant therapy in the future to prompt changes in systemic regimens.

## Conclusion

The treatment of patients with stage IIIA (*N*2) NSCLC is complex and requires the expertise of a multidisciplinary thoracic oncology team. Neoadjuvant platinum-based chemotherapy followed by surgery has been found to improve survival in patients with locally advanced NSCLC (28–31). Neoadjuvant chemoradiation improves nodal clearance (33) and is a strong rationale for adding radiation to the neoadjuvant treatment approach. Although improved survival with neoadjuvant chemoradiation followed by resection has not been shown in phase III trials to date (33), these studies have been limited by slow accrual rates, patient selection, outdated radiation techniques, detrimental interruptions in therapy, and high mortality rates associated with pneumonectomy.

The benefit of surgery following neoadjuvant treatment in comparison to definitive concurrent chemoradiation remains unclear based on currently available phase III data. However, there is evidence to suggest that an appropriately selected, rigorously screened subset of stage IIIA patients with *N*2 disease may experience a survival benefit from a tri-modality approach of surgical resection after neoadjuvant concurrent chemoradiation (34). It is critical that a thoracic surgeon evaluates these patients prior to initiating therapy, and if a lobectomy with complete resection (R0) can be performed with reasonable certainty, chemoradiation is a reasonable neoadjuvant option. Similar to induction chemotherapy, chemotherapy in the tri-modality setting should be platinum-based, preferably with cisplatin, although carboplatin is an option in patients who cannot receive cisplatin-containing regimens. Furthermore, care should be taken to minimize interruptions in therapy, even for restaging, which can lead to suboptimal treatment strategies and potentially inferior outcomes.

Finally, targeted therapy and immunotherapy are the major areas of current clinical trial research, and it is expected that accrual rates will improve now that immunotherapy has stolen center stage of neoadjuvant clinical trial design for NSCLC. The results from these trials will hopefully lead to additional systemic options to further improve upon the cure rate for our future patients with stage IIIA (*N*2) NSCLC.

## Author Contributions

LH and JL are responsible for the conception of the article. JL is responsible for the initial content. JL, LH, EG, and EO are responsible for the intellectual content, critical analysis, and revisions of the manuscript.

## Conflict of Interest Statement

The authors declare that the research was conducted in the absence of any commercial or financial relationships that could be construed as a potential conflict of interest. The reviewer MP and handling editor declared their shared affiliation.

## References

[1] American Cancer Soceity. Cancer Facts & Figures 2017. Atlanta: American Cancer Society (2017).

[2] van Meerbeeck JP Kramer GW Van Schil PE Legrand C Smit EF Schramel F Randomized controlled trial of resection versus radiotherapy after induction chemotherapy in stage IIIA-N2 non-small-cell lung cancer. J Natl Cancer Inst (2007) 99(6):442–50. 10.1093/jnci/djk093 17374834

[3] Andre F Grunenwald D Pignon JP Dujon A Pujol JL Brichon PY Survival of patients with resected N2 non-small-cell lung cancer: evidence for a subclassification and implications. J Clin Oncol (2000) 18(16):2981–9. 10.1200/JCO.2000.18.16.2981 10944131

[4] Katsuki H Shimada K Koyama A Okita M Yamaguchi Y. Long-term intermittent adjuvant chemotherapy for primary, resected lung cancer. J Thorac Cardiovasc Surg (1975) 70(4):590–605. 1177474

[5] Martini N Flehinger BJ. The role of surgery in N2 lung cancer. Surg Clin North Am (1987) 67(5):1037–49. 10.1016/S0039-6109(16)44341-0 3629423

[6] Martini N Flehinger BJ Zaman MB Beattie EJ Jr Prospective study of 445 lung carcinomas with mediastinal lymph node metastases. J Thorac Cardiovasc Surg (1980) 80(3):390–9. 6251316

[7] Bains MS Surgical treatment of lung cancer. Chest (1991) 100(3):826–37. 10.1378/chest.100.3.826 1889280

[8] Johnson DH Piantadosi S Chemotherapy for resectable stage III non-small-cell lung cancer – can that dog hunt? J Natl Cancer Inst (1994) 86(9):650–1. 10.1093/jnci/86.9.650 8158690

[9] Farray D Mirkovic N Albain KS. Multimodality therapy for stage III non-small-cell lung cancer. J Clin Oncol (2005) 23(14):3257–69. 10.1200/JCO.2005.03.008 15886313

[10] Martini N Kris MG Gralla RJ Bains MS McCormack PM Kaiser LR The effects of preoperative chemotherapy on the resectability of non-small cell lung carcinoma with mediastinal lymph node metastases (N2 M0). Ann Thorac Surg (1988) 45(4):370–9. 10.1016/S0003-4975(98)90007-8 2833188

[11] Vokes EE Bitran JD Hoffman PC Ferguson MK Weichselbaum RR Golomb HM Neoadjuvant vindesine, etoposide, and cisplatin for locally advanced non-small cell lung cancer. Final report of a phase 2 study. Chest (1989) 96(1):110–3. 2544349 10.1378/chest.96.1.110

[12] Pujol JL Rossi JF Le Chevalier T Daures JP Rouanet P Douillard JY Pilot study of neoadjuvant ifosfamide, cisplatin, and etoposide in locally advanced non-small cell lung cancer. Eur J Cancer (1990) 26(7):798–801. 10.1016/0277-5379(90)90155-M 2171600

[13] Burkes RL Ginsberg RJ Shepherd FA Blackstein ME Goldberg ME Waters PF Induction chemotherapy with mitomycin, vindesine, and cisplatin for stage III unresectable non-small-cell lung cancer: results of the Toronto Phase II Trial. J Clin Oncol (1992) 10(4):580–6. 10.1200/JCO.1992.10.4.580 1312587

[14] Martini N Kris MG Flehinger BJ Gralla RJ Bains MS Burt ME Preoperative chemotherapy for stage IIIa (N2) lung cancer: the Sloan-Kettering experience with 136 patients. Ann Thorac Surg (1993) 55(6):1365–73; discussion 73–4. 10.1016/0003-4975(93)91072-U 8390230

[15] Darwish S Minotti V Crino L Rossetti R Maranzano E Checcaglini F Neoadjuvant cisplatin and etoposide for stage IIIA (clinical N2) non-small cell lung cancer. Am J Clin Oncol (1994) 17(1):64–7. 10.1097/00000421-199402000-00014 8311011

[16] Sugarbaker DJ Herndon J Kohman LJ Krasna MJ Green MR. Results of cancer and leukemia group B protocol 8935. A multiinstitutional phase II trimodality trial for stage IIIA (N2) non-small-cell lung cancer. Cancer and Leukemia Group B Thoracic Surgery Group. J Thorac Cardiovasc Surg (1995) 109(3):473–83; discussion 83–5. 10.1016/S0022-5223(95)70278-4 7877308

[17] Elias AD Skarin AT Leong T Mentzer S Strauss G Lynch T Neoadjuvant therapy for surgically staged IIIA N2 non-small cell lung cancer (NSCLC). Lung Cancer (1997) 17(1):147–61. 10.1016/S0169-5002(97)00658-2 9194034

[18] Van Zandwijk N Smit EF Kramer GW Schramel F Gans S Festen J Gemcitabine and cisplatin as induction regimen for patients with biopsy-proven stage IIIA N2 non-small-cell lung cancer: a phase II study of the European Organization for Research and Treatment of Cancer Lung Cancer Cooperative Group (EORTC 08955). J Clin Oncol (2000) 18(14):2658–64. 10.1200/JCO.2000.18.14.2658 10894864

[19] Betticher DC Hsu Schmitz SF Totsch M Hansen E Joss C von Briel C Mediastinal lymph node clearance after docetaxel-cisplatin neoadjuvant chemotherapy is prognostic of survival in patients with stage IIIA pN2 non-small-cell lung cancer: a multicenter phase II trial. J Clin Oncol (2003) 21(9):1752–9. 10.1200/JCO.2003.11.040 12721251

[20] O’Brien ME Splinter T Smit EF Biesma B Krzakowski M Tjan-Heijnen VC Carboplatin and paclitaxol (Taxol) as an induction regimen for patients with biopsy-proven stage IIIA N2 non-small cell lung cancer. an EORTC phase II study (EORTC 08958). Eur J Cancer (2003) 39(10):1416–22. 10.1016/S0959-8049(03)00319-8 12826045

[21] De Marinis F Nelli F Migliorino MR Martelli O Cortesi E Treggiari S Gemcitabine, paclitaxel, and cisplatin as induction chemotherapy for patients with biopsy-proven stage IIIA(N2) nonsmall cell lung carcinoma: a phase II multicenter study. Cancer (2003) 98(8):1707–15. 10.1002/cncr.11662 14534888

[22] Cappuzzo F Selvaggi G Gregorc V Mazzoni F Betti M Rita Migliorino M Gemcitabine and cisplatin as induction chemotherapy for patients with unresectable stage IIIA-bulky N2 and stage IIIB nonsmall cell lung carcinoma: an Italian Lung Cancer Project Observational Study. Cancer (2003) 98(1):128–34. 10.1002/cncr.11460 12833465

[23] Burkes RL Shepherd FA Blackstein ME Goldberg ME Waters PF Patterson GA Induction chemotherapy with mitomycin, vindesine, and cisplatin for stage IIIA (T1-3, N2) unresectable non-small-cell lung cancer: final results of the Toronto phase II trial. Lung Cancer (2005) 47(1):103–9. 10.1016/j.lungcan.2004.06.004 15603860

[24] Biesma B Manegold C Smit HJ Willems L Legrand C Passioukov A Docetaxel and cisplatin as induction chemotherapy in patients with pathologically-proven stage IIIA N2 non-small cell lung cancer: a phase II study of the European organization for research and treatment of cancer (EORTC 08984). Eur J Cancer (2006) 42(10):1399–406. 10.1016/j.ejca.2006.01.049 16759850

[25] Garrido P Gonzalez-Larriba JL Insa A Provencio M Torres A Isla D Long-term survival associated with complete resection after induction chemotherapy in stage IIIA (N2) and IIIB (T4N0-1) non small-cell lung cancer patients: the Spanish Lung Cancer Group Trial 9901. J Clin Oncol (2007) 25(30):4736–42. 10.1200/JCO.2007.12.0014 17947721

[26] Chaft JE Rusch V Ginsberg MS Paik PK Finley DJ Kris MG Phase II trial of neoadjuvant bevacizumab plus chemotherapy and adjuvant bevacizumab in patients with resectable nonsquamous non-small-cell lung cancers. J Thorac Oncol (2013) 8(8):1084–90. 10.1097/JTO.0b013e31829923ec 23857398 PMC4191830

[27] Ou W Li N Wang SY Li J Liu QW Huang QA Phase 2 trial of neoadjuvant bevacizumab plus pemetrexed and carboplatin in patients with unresectable stage III lung adenocarcinoma (GASTO 1001). Cancer (2016) 122(5):740–7. 10.1002/cncr.29800 26700505

[28] Pass HI Pogrebniak HW Steinberg SM Mulshine J Minna J. Randomized trial of neoadjuvant therapy for lung cancer: interim analysis. Ann Thorac Surg (1992) 53(6):992–8. 10.1016/0003-4975(92)90373-C 1317697

[29] Rosell R Gomez-Codina J Camps C Maestre J Padille J Canto A A randomized trial comparing preoperative chemotherapy plus surgery with surgery alone in patients with non-small-cell lung cancer. N Engl J Med (1994) 330(3):153–8. 10.1056/NEJM199401203300301 8043059

[30] Roth JA Fossella F Komaki R Ryan MB Putnam JB Jr Lee JS A randomized trial comparing perioperative chemotherapy and surgery with surgery alone in resectable stage IIIA non-small-cell lung cancer. J Natl Cancer Inst (1994) 86(9):673–80. 10.1093/jnci/86.9.673 8158698

[31] Depierre A Milleron B Moro-Sibilot D Chevret S Quoix E Lebeau B Preoperative chemotherapy followed by surgery compared with primary surgery in resectable stage I (except T1N0), II, and IIIa non-small-cell lung cancer. J Clin Oncol (2002) 20(1):247–53. 10.1200/JCO.20.1.247 11773176

[32] Burdett S Stewart LA Rydzewska L. A systematic review and meta-analysis of the literature: chemotherapy and surgery versus surgery alone in non-small cell lung cancer. J Thorac Oncol (2006) 1(7):611–21. 10.1016/S1556-0864(15)30371-3 17409927

[33] Thomas M Rube C Hoffknecht P Macha HN Freitag L Linder A Effect of preoperative chemoradiation in addition to preoperative chemotherapy: a randomised trial in stage III non-small-cell lung cancer. Lancet Oncol (2008) 9(7):636–48. 10.1016/S1470-2045(08)70156-6 18583190

[34] Albain KS Swann RS Rusch VW Turrisi AT III Shepherd FA Smith C Radiotherapy plus chemotherapy with or without surgical resection for stage III non-small-cell lung cancer: a phase III randomised controlled trial. Lancet (2009) 374(9687):379–86. 10.1016/S0140-6736(09)60737-6 19632716 PMC4407808

[35] Palazzi M Cataldo I Gramaglia A De Toma D Milani F Ravasi G. Preoperative concomitant cisplatin/VP16 and radiotherapy in stage III non-small cell lung cancer. Int J Radiat Oncol Biol Phys (1993) 27(3):621–5. 10.1016/0360-3016(93)90388-C 8226157

[36] Favaretto A Paccagnella A Tomio L Sartori F Cipriani A Zuin R Pre-operative chemoradiotherapy in non-small cell lung cancer stage III patients. Feasibility, toxicity and long-term results of a phase II study. Eur J Cancer (1996) 32A(12):2064–9. 10.1016/S0959-8049(96)00248-1 9014746

[37] Albain KS Rusch VW Crowley JJ Rice TW Turrisi AT III Weick JK Concurrent cisplatin/etoposide plus chest radiotherapy followed by surgery for stages IIIA (N2) and IIIB non-small-cell lung cancer: mature results of Southwest Oncology Group phase II study 8805. J Clin Oncol (1995) 13(8):1880–92. 10.1200/JCO.1995.13.8.1880 7636530

[38] Eberhardt W Wilke H Stamatis G Stuschke M Harstrick A Menker H Preoperative chemotherapy followed by concurrent chemoradiation therapy based on hyperfractionated accelerated radiotherapy and definitive surgery in locally advanced non-small-cell lung cancer: mature results of a phase II trial. J Clin Oncol (1998) 16(2):622–34. 10.1200/JCO.1998.16.2.622 9469351

[39] Winton T Livingston R Johnson D Rigas J Johnston M Butts C Vinorelbine plus cisplatin vs. observation in resected non-small-cell lung cancer. N Engl J Med (2005) 352(25):2589–97. 10.1056/NEJMoa043623 15972865

[40] Kreuter M Vansteenkiste J Fischer JR Eberhardt W Zabeck H Kollmeier J Randomized phase 2 trial on refinement of early-stage NSCLC adjuvant chemotherapy with cisplatin and pemetrexed versus cisplatin and vinorelbine: the TREAT study. Ann Oncol (2013) 24(4):986–92. 10.1093/annonc/mds578 23161898

[41] Gilligan D Nicolson M Smith I Groen H Dalesio O Goldstraw P Preoperative chemotherapy in patients with resectable non-small cell lung cancer: results of the MRC LU22/NVALT 2/EORTC 08012 multicentre randomised trial and update of systematic review. Lancet (2007) 369(9577):1929–37. 10.1016/S0140-6736(07)60714-4 17544497

[42] Pisters KM Vallieres E Crowley JJ Franklin WA Bunn PA Jr Ginsberg RJ Surgery with or without preoperative paclitaxel and carboplatin in early-stage non-small-cell lung cancer: Southwest Oncology Group Trial S9900, an intergroup, randomized, phase III trial. J Clin Oncol (2010) 28(11):1843–9. 10.1200/JCO.2009.26.1685 20231678 PMC2860367

[43] Felip E Rosell R Maestre JA Rodriguez-Paniagua JM Moran T Astudillo J Preoperative chemotherapy plus surgery versus surgery plus adjuvant chemotherapy versus surgery alone in early-stage non-small-cell lung cancer. J Clin Oncol (2010) 28(19):3138–45. 10.1200/JCO.2009.27.6204 20516435

[44] Evans NR III Li S Wright CD Allen MS Gaissert HA. The impact of induction therapy on morbidity and operative mortality after resection of primary lung cancer. J Thorac Cardiovasc Surg (2010) 139(4):991–6.e1–2. 10.1016/j.jtcvs.2009.11.070 20304144

[45] Mansour Z Kochetkova EA Ducrocq X Vasilescu MD Maxant G Buggenhout A Induction chemotherapy does not increase the operative risk of pneumonectomy! Eur J Cardiothorac Surg (2007) 31(2):181–5. 10.1016/j.ejcts.2006.11.008 17141515

[46] Stefani A Alifano M Bobbio A Grigoroiu M Jouni R Magdeleinat P Which patients should be operated on after induction chemotherapy for N2 non-small cell lung cancer? Analysis of a 7-year experience in 175 patients. J Thorac Cardiovasc Surg (2010) 140(2):356–63. 10.1016/j.jtcvs.2010.02.018 20381815

[47] Taylor SG Trybula M Bonomi PD Faber LP Lee MS Reddy S Simultaneous cisplatin fluorouracil infusion and radiation followed by surgical resection in regionally localized stage III, non-small cell lung cancer. Ann Thorac Surg (1987) 43(1):87–91. 10.1016/S0003-4975(10)60173-7 3026263

[48] Pincus M Reddy S Lee MS Bonomi P Taylor S Rowland K Preoperative combined modality therapy for stage III M0 non-small cell lung carcinoma. Int J Radiat Oncol Biol Phys (1988) 15(1):189–95. 10.1016/0360-3016(88)90365-3 2839440

[49] Faber LP Kittle CF Warren WH Bonomi PD Taylor SG Reddy S Preoperative chemotherapy and irradiation for stage III non-small cell lung cancer. Ann Thorac Surg (1989) 47(5):669–75; discussion 76–7. 10.1016/0003-4975(89)90115-X 2543340

[50] Recine D Rowland K Reddy S Lee MS Bonomi P Taylor S Combined modality therapy for locally advanced non-small cell lung carcinoma. Cancer (1990) 66(11):2270–8. 10.1002/1097-0142(19901201)66:11<2270::AID-CNCR2820661104>3.0.CO;2-H 2173969

[51] Strauss GM Herndon JE Sherman DD Mathisen DJ Carey RW Choi NC Neoadjuvant chemotherapy and radiotherapy followed by surgery in stage IIIA non-small-cell carcinoma of the lung: report of a Cancer and Leukemia Group B phase II study. J Clin Oncol (1992) 10(8):1237–44. 10.1200/JCO.1992.10.8.1237 1321893

[52] Weiden PL Piantadosi S. Preoperative chemotherapy (cisplatin and fluorouracil) and radiation therapy in stage III non-small cell lung cancer. A phase 2 study of the LCSG. Chest (1994) 106(6 Suppl):344S–7S. 10.1378/chest.106.6.344S 7988261

[53] Choi NC Carey RW Daly W Mathisen D Wain J Wright C Potential impact on survival of improved tumor downstaging and resection rate by preoperative twice-daily radiation and concurrent chemotherapy in stage IIIA non-small-cell lung cancer. J Clin Oncol (1997) 15(2):712–22. 10.1200/JCO.1997.15.2.712 9053497

[54] Thomas M Rube C Semik M von Eiff M Freitag L Macha HN Impact of preoperative bimodality induction including twice-daily radiation on tumor regression and survival in stage III non-small-cell lung cancer. J Clin Oncol (1999) 17(4):1185. 10.1200/JCO.1999.17.4.1185 10561177

[55] D’Angelillo RM Trodella L Ciresa M Cellini F Fiore M Greco C Multimodality treatment of stage III non-small cell lung cancer: analysis of a phase II trial using preoperative cisplatin and gemcitabine with concurrent radiotherapy. J Thorac Oncol (2009) 4(12):1517–23. 10.1097/JTO.0b013e3181b9e860 19875976

[56] Shah AA Berry MF Tzao C Gandhi M Worni M Pietrobon R Induction chemoradiation is not superior to induction chemotherapy alone in stage IIIA lung cancer. Ann Thorac Surg (2012) 93(6):1807–12. 10.1016/j.athoracsur.2012.03.018 22632486

[57] Sher DJ Fidler MJ Liptay MJ Koshy M. Comparative effectiveness of neoadjuvant chemoradiotherapy versus chemotherapy alone followed by surgery for patients with stage IIIA non-small cell lung cancer. Lung Cancer (2015) 88(3):267–74. 10.1016/j.lungcan.2015.03.015 25862147

[58] Higgins K Chino JP Marks LB Ready N D’Amico TA Clough RW Preoperative chemotherapy versus preoperative chemoradiotherapy for stage III (N2) non-small-cell lung cancer. Int J Radiat Oncol Biol Phys (2009) 75(5):1462–7. 10.1016/j.ijrobp.2009.01.069 19467798

[59] Decaluwe H De Leyn P Vansteenkiste J Dooms C Van Raemdonck D Nafteux P Surgical multimodality treatment for baseline resectable stage IIIA-N2 non-small cell lung cancer. Degree of mediastinal lymph node involvement and impact on survival. Eur J Cardiothorac Surg (2009) 36(3):433–9. 10.1016/j.ejcts.2009.04.013 19502079

[60] Cerfolio RJ Maniscalco L Bryant AS. The treatment of patients with stage IIIA non-small cell lung cancer from N2 disease: who returns to the surgical arena and who survives. Ann Thorac Surg (2008) 86(3):912–20; discussion 20. 10.1016/j.athoracsur.2008.04.073 18721582

[61] Auperin A Le Pechoux C Rolland E Curran WJ Furuse K Fournel P Meta-analysis of concomitant versus sequential radiochemotherapy in locally advanced non-small-cell lung cancer. J Clin Oncol (2010) 28(13):2181–90. 10.1200/JCO.2009.26.2543 20351327

[62] Chen M Jiang GL Fu XL Wang LJ Qian H Chen GY The impact of overall treatment time on outcomes in radiation therapy for non-small cell lung cancer. Lung Cancer (2000) 28(1):11–9. 10.1016/S0169-5002(99)00113-0 10704704

[63] Bese NS Hendry J Jeremic B. Effects of prolongation of overall treatment time due to unplanned interruptions during radiotherapy of different tumor sites and practical methods for compensation. Int J Radiat Oncol Biol Phys (2007) 68(3):654–61. 10.1016/j.ijrobp.2007.03.010 17467926

[64] Martins RG D’Amico TA Loo BW Jr Pinder-Schenck M Borghaei H Chaft JE The management of patients with stage IIIA non-small cell lung cancer with N2 mediastinal node involvement. J Natl Compr Canc Netw (2012) 10(5):599–613. 10.6004/jnccn.2012.0062 22570291

[65] Douillard JY Rosell R De Lena M Riggi M Hurteloup P Mahe MA Impact of postoperative radiation therapy on survival in patients with complete resection and stage I, II, or IIIA non-small-cell lung cancer treated with adjuvant chemotherapy: the adjuvant Navelbine International Trialist Association (ANITA) Randomized Trial. Int J Radiat Oncol Biol Phys (2008) 72(3):695–701. 10.1016/j.ijrobp.2008.01.044 18439766

[66] Burdett S Rydzewska L Tierney J Fisher D Parmar MK Arriagada R Postoperative radiotherapy for non-small cell lung cancer. Cochrane Database Syst Rev (2016) 10:CD002142. 10.1002/14651858.CD002142.pub4 27727451 PMC5642866

[67] Belani CP Choy H Bonomi P Scott C Travis P Haluschak J Combined chemoradiotherapy regimens of paclitaxel and carboplatin for locally advanced non-small-cell lung cancer: a randomized phase II locally advanced multi-modality protocol. J Clin Oncol (2005) 23(25):5883–91. 10.1200/JCO.2005.55.405 16087941

[68] Ohe Y Ohashi Y Kubota K Tamura T Nakagawa K Negoro S Randomized phase III study of cisplatin plus irinotecan versus carboplatin plus paclitaxel, cisplatin plus gemcitabine, and cisplatin plus vinorelbine for advanced non-small-cell lung cancer: Four-Arm Cooperative Study in Japan. Ann Oncol (2007) 18(2):317–23. 10.1093/annonc/mdl377 17079694

[69] Senan S Brade A Wang LH Vansteenkiste J Dakhil S Biesma B PROCLAIM: randomized phase III trial of pemetrexed-cisplatin or etoposide-cisplatin plus thoracic radiation therapy followed by consolidation chemotherapy in locally advanced nonsquamous non-small-cell lung cancer. J Clin Oncol (2016) 34(9):953–62. 10.1200/JCO.2015.64.8824 26811519

[70] ClinialTrials.gov. (2017). Available from: https://clinicaltrials.gov/ct2/results?cond=NSCLC%2C+Stage+IIIA&term=chemoradiation&cntry=&state=&city=&dist=

[71] Liang J Bi N Wu S Chen M Lv C Zhao L Etoposide and cisplatin versus paclitaxel and carboplatin with concurrent thoracic radiotherapy in unresectable stage III non-small cell lung cancer: a multicenter randomized phase III trial. Ann Oncol (2017) 28(4):777–83. 10.1093/annonc/mdx009 28137739

[72] Kramer GW Legrand CL van Schil P Uitterhoeve L Smit EF Schramel F Quality assurance of thoracic radiotherapy in EORTC 08941: a randomised trial of surgery versus thoracic radiotherapy in patients with stage IIIA non-small-cell lung cancer (NSCLC) after response to induction chemotherapy. Eur J Cancer (2006) 42(10):1391–8. 10.1016/j.ejca.2006.01.052 16785054

[73] Bradley JD Paulus R Komaki R Masters G Blumenschein G Schild S Standard-dose versus high-dose conformal radiotherapy with concurrent and consolidation carboplatin plus paclitaxel with or without cetuximab for patients with stage IIIA or IIIB non-small-cell lung cancer (RTOG 0617): a randomised, two-by-two factorial phase 3 study. Lancet Oncol (2015) 16(2):187–99. 10.1016/S1470-2045(14)71207-0 25601342 PMC4419359

[74] Suntharalingam M Paulus R Edelman MJ Krasna M Burrows W Gore E Radiation therapy oncology group protocol 02-29: a phase II trial of neoadjuvant therapy with concurrent chemotherapy and full-dose radiation therapy followed by surgical resection and consolidative therapy for locally advanced non-small cell carcinoma of the lung. Int J Radiat Oncol Biol Phys (2012) 84(2):456–63. 10.1016/j.ijrobp.2011.11.069 22543206

[75] Samson P Crabtree TD Robinson CG Morgensztern D Broderick S Krupnick AS Defining the ideal time interval between planned induction therapy and surgery for stage IIIA non-small cell lung cancer. Ann Thorac Surg (2017) 103(4):1070–5. 10.1016/j.athoracsur.2016.09.053 28110809 PMC5444908

[76] Gao SJ Corso CD Wang EH Blasberg JD Detterbeck FC Boffa DJ Timing of surgery after neoadjuvant chemoradiation in locally advanced non-small cell lung cancer. J Thorac Oncol (2017) 12(2):314–22. 10.1016/j.jtho.2016.09.122 27720827

[77] Zhong W Yang X Yan H Zhang X Su J Chen Z Phase II study of biomarker-guided neoadjuvant treatment strategy for IIIA-N2 non-small cell lung cancer based on epidermal growth factor receptor mutation status. J Hematol Oncol (2015) 8:54. 10.1186/s13045-015-0151-3 25981169 PMC4455050

[78] Schaake EE Kappers I Codrington HE Valdes Olmos RA Teertstra HJ van Pel R Tumor response and toxicity of neoadjuvant erlotinib in patients with early-stage non-small-cell lung cancer. J Clin Oncol (2012) 30(22):2731–8. 10.1200/JCO.2011.39.4882 22753915

[79] ClinicalTrials.gov. (2017). Available from: https://www.clinicaltrials.gov/ct2/results?cond=Lung+Cancer+Neoadjuvant&term=&cntry1=&state1=&Search=Search

[80] Juweid ME Cheson BD Positron-emission tomography and assessment of cancer therapy. N Engl J Med (2006) 354(5):496–507. 10.1056/NEJMra050276 16452561

[81] Berghmans T Dusart M Paesmans M Hossein-Foucher C Buvat I Castaigne C Primary tumor standardized uptake value (SUVmax) measured on fluorodeoxyglucose positron emission tomography (FDG-PET) is of prognostic value for survival in non-small cell lung cancer (NSCLC): a systematic review and meta-analysis (MA) by the European Lung Cancer Working Party for the IASLC Lung Cancer Staging Project. J Thorac Oncol (2008) 3(1):6–12. 10.1097/JTO.0b013e31815e6d6b 18166834

[82] Bharat AGB Rusch VW Bains MS Rizk NP Role of PET scan in predicting response to neoadjuvant chemotherapy and long-term outcomes for stage II lung cancer. ASCO Meet Abstr (2014) 32(15_suppl):7574 10.1200/jco.2014.32.15_suppl.7574

[83] Hoekstra CJ Stroobants SG Smit EF Vansteenkiste J van Tinteren H Postmus PE Prognostic relevance of response evaluation using [18F]-2-fluoro-2-deoxy-D-glucose positron emission tomography in patients with locally advanced non-small-cell lung cancer. J Clin Oncol (2005) 23(33):8362–70. 10.1200/JCO.2005.01.1189 16293866

[84] Chaft JE Dunphy M Naidoo J Travis WD Hellmann M Woo K Adaptive neoadjuvant chemotherapy guided by (18)F-FDG PET in resectable non-small cell lung cancers: the NEOSCAN trial. J Thorac Oncol (2016) 11(4):537–44. 10.1016/j.jtho.2015.12.104 26724474 PMC4808609

